# Application of Bacteriophages in the Agro-Food Sector: A Long Way Toward Approval

**DOI:** 10.3389/fcimb.2018.00296

**Published:** 2018-08-22

**Authors:** Lucía Fernández, Diana Gutiérrez, Ana Rodríguez, Pilar García

**Affiliations:** Departamento de Tecnología y Biotecnología de Productos Lácteos, Instituto de Productos Lácteos de Asturias (IPLA-CSIC), Villaviciosa, Spain

**Keywords:** bacteriophage, phage lytic proteins, phage therapy, food industry, disinfection

The rapid increase in multi-drug resistant (MDR) pathogens has put notable pressure on Health Authorities, who foresee an uncertain future for global human health. In June 2017, the European Commission adopted the “EU One Health action plan against antimicrobial resistance,” thereby providing a framework for reducing the emergence and spread of antimicrobial resistance as well as boosting the development of new effective antimicrobials. Nowadays, nobody doubts that the use of antibiotics in agro-food production should be properly controlled, as it represents a major source of bacterial resistance acquisition that further reaches clinical settings. Therefore, bacteria control measures other than antibiotics are presently being assessed in order to both reduce food-borne outbreaks and stop the spread of MDR zoonotic bacteria. Among the many antimicrobials suggested to replace or complement traditional antibiotics, bacteriophages and phage-derived proteins (collectively known as phage therapy) are strong candidates for the treatment of human bacterial infections, rescuing the idea from previous work made in Eastern Europe (Sulakvelidze and Morris, 2001). However, after their resurgence almost 20 years ago, it is rather puzzling that we do not have more commercial phage-based products to fight against MDR bacteria. To understand why phage therapy strategies are taking so long to reach the market, we need to examine in detail the nature and properties of these antimicrobials, as well as the existing legal framework.

While bacteriophages can be rightfully considered the natural enemies of bacteria, these viruses also maintain an important relationship with their hosts through modulating microbial populations and promoting their evolution by horizontal gene transfer (Clokie et al., 2011). Indeed, as the most abundant organisms in the biosphere, the role of phages in different ecosystems, such as the oceans (Breitbart, 2012) or the human gut, is generating a huge interest (Manrique et al., 2017). As antimicrobials, phages offer some compelling advantages over more conventional agents, including specificity, safety, effectiveness against MDR bacteria, and easy genetic manipulation. Moreover, phage lytic enzymes (endolysins and virion-associated peptidoglycan hydrolases) are considered as a new class of antibiotics, named enzybiotics, and also exhibit many desirable properties. Thus, in addition to high lytic ability, specificity and modular structure, it is worth noting that bacteria resistant to enzybiotics have not been detected so far (Fischetti, 2010; Rodríguez-Rubio et al., 2013).

Following publication of the early successful results of phage therapy, the idea was quickly translated to other fields such as agriculture, veterinary medicine, food safety, and wastewater treatment, where this approach was well received (García et al., 2010; Jassim et al., 2016; Carvalho et al., 2017; Figure 1). In the agro-food context, both types of antimicrobials (phages and phage lytic proteins) can be used to ensure food safety along the whole food chain (from farm to fork) (García et al., 2008). One potential target sector for the application of bacteriophages is primary production, where they can replace or complement antibiotics and pesticides. As a result, phages could help to limit the rise in antibiotic resistance associated with agricultural and livestock farming practices. Indeed, bacteriophages have already given successful results against bacterial crop diseases, and three different phage-based products (Agriphage in the US, Erwiphage in Hungary, and Biolyse in the UK) are currently on the market despite limitations due to the sensitivity of phages to UV

**Figure 1:**
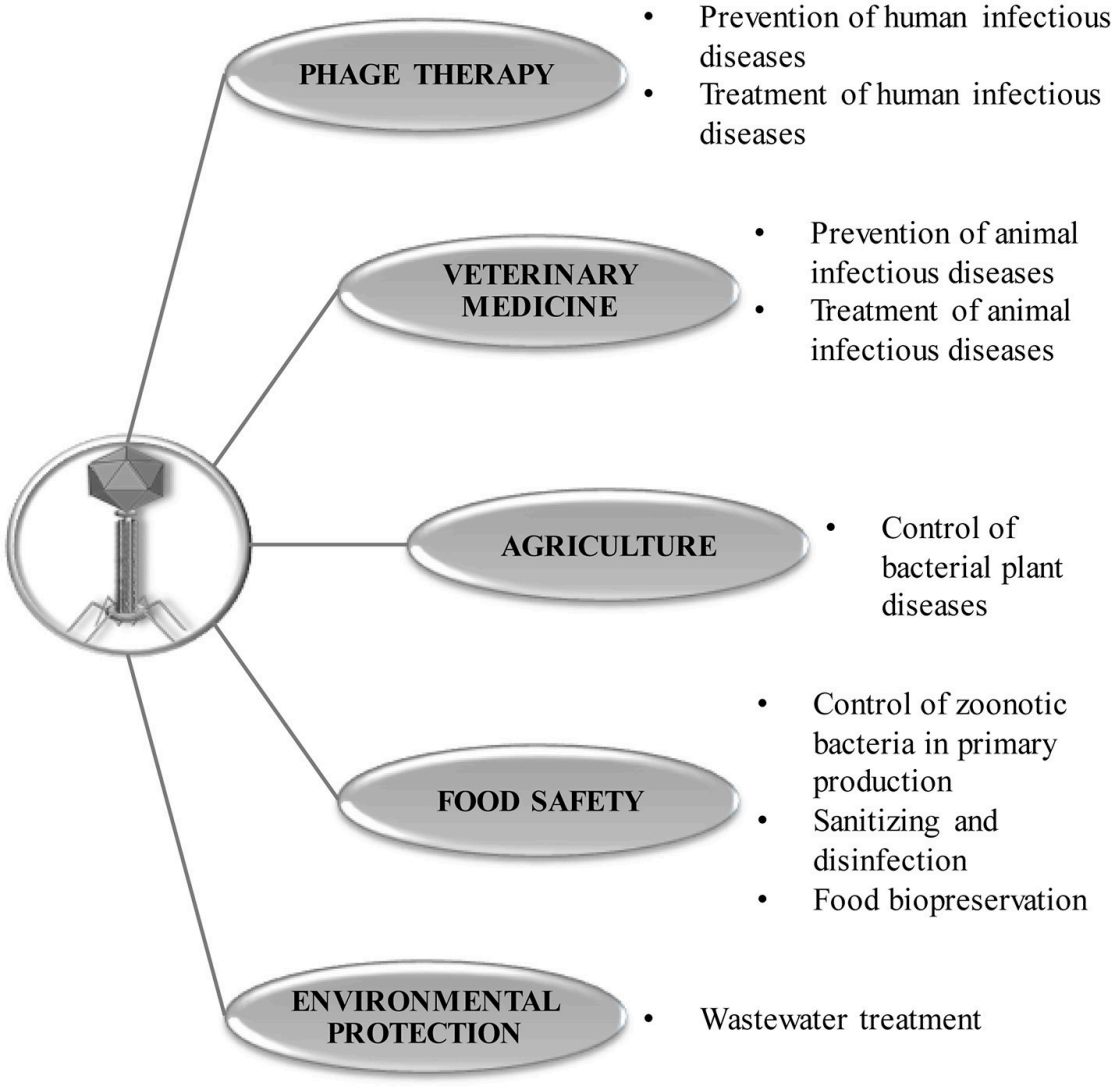
Current applications of phages and phage-derived lytic proteins.

light or certain soil conditions (Buttimer et al., 2017). The other large sector in primary production includes livestock farming and aquaculture, where the need for antibiotics is expected to continue increasing dramatically to meet the growing demand for meat and fish (Van Boeckel et al., 2015). The use of bacteriophages to reduce zoonotic bacteria in animals is promising (Atterbury, 2009), with the main drawbacks being inactivation of phages in the gut and removal of phages from the circulatory system following oral and parenteral administration, respectively. However, there are several studies demonstrating the efficacy and potential of phage therapy in animals for the control of infectious diseases (Richards, 2014; Carvalho et al., 2017; Doss et al., 2017; Wernicki et al., 2017). Furthermore, a US company has developed and licensed bacteriophage-based animal healthcare products effective against *Salmonella* (PLSV-1™) and *Clostridium perfringens* (INT-401™) in poultry, aimed at both prophylaxis and treatment.

Although more indirectly, surface sanitizing in livestock facilities and food industries also constitutes a source of antimicrobial resistance emergence due to the existence of cross-resistance between biocides and antibiotics (Hernández et al., 2011). In this regard, phages can also provide an attractive alternative to traditional disinfectants and some products have already been approved for this purpose. For example, two US companies commercialize products for the disinfection of surfaces against *Salmonella* (BacWash™) and *E. coli* O157:H7 (Finalyse™). Besides surface decontamination, phages can also be applied as food biopreservatives. Although this application appears even more complicated from a regulatory perspective, several phage products intended as food additives are currently marketed in the US. Thus, phage preparations against *Listeria monocytogenes* (Listshield™), *Salmonella enterica* (SalmoFresh™), and *Escherichia coli* (Ecoshield™) have received GRAS designation by the FDA for direct application to food and are commercially available. In the EU, on the other hand, the use of bacteriophages in food from animal origin was evaluated by the European Food Safety Authority (EFSA) in 2009. More recently, the use of PhageGuard Listex for removing *L. monocytogenes* from raw fish was assessed in 2012. In both studies, EFSA concluded that bacteriophages are harmless for consumers but it is not clear whether they can protect against food re-contamination. Finally, bacteriophages have been approved as processing-aids in food processing and handling in several countries. Indeed, two such products against *L. monocytogenes* (PhageGuard Listex) and *Salmonella* (PhageGuard S) are currently available. Both products can also be used for decontamination of surfaces.

Overall, the main hurdles for the extensive use of bacteriophages in the agro-food industry can be summarized in technical and legal inconveniences. From a legal perspective, regulatory frameworks for antimicrobials are very restrictive in most countries, seriously limiting the introduction of new compounds in the agro-food sector.

Regarding technical shortcomings, some of the problems encountered affect phage stability, bacterial resistance or gene transfer, many of which can potentially be solved in a relatively easy way with the current engineering techniques e.g., CRISPR-Cas (Bari et al., 2017). However, further work is still necessary to adapt lab-scale results to large-scale bacteriophage production and purification by companies according to good manufacturing practices (GMP). Another important drawback regarding the commercialization of phage-based products is the need to design phage mixtures effective against the highest possible number of strains belonging to the target bacterium. Additionally, these mixtures often have to be regularly adapted to avoid the emergence of phage-resistant strains in the place of application. Moreover, this disadvantage is frequently aggravated by the existence of restrictive regulations. For instance, in the EU, changes in the phage mixture composition of a plant protection product may require a new registration (1107/2009 EC).

In addition to the setbacks indicated above, there are other unsolved concerns such as the consequences of introducing high concentrations of phages in the environment. In that sense, it is worth analysing carefully the previous mistakes made with antibiotics. For example, the scientific community is now aware that the possibility of selecting multi-phage resistant bacteria, the evolution of the target bacteria or how phages may affect microbial communities in nature should be evaluated during the development of novel antimicrobial strategies. The frequency of phage resistance development can vary depending on the specific phage between 10^−4^ and 10^−7^ (Gutiérrez et al., 2015; Silva et al., 2016; Valério et al., 2017). However, it must be mentioned that this frequency can be 10- or even 100-fold lower than that of antibiotic resistance development (Carlton, 1999; Valério et al., 2017). Moreover, there are some interesting characteristics of phage resistance that make it different from antibiotic resistance and are, as a result, also worth mentioning here (Oechslin, 2018). Most importantly, phage resistance would only arise in cells of the target species and it is generally the result of mutations and, consequently, non-transmissible. Moreover, these mutations frequently affect phage receptor proteins which often play essential roles for the bacterium, thereby leading to fitness and/or virulence loss. Finally, unlike other antimicrobials, bacteriophages can themselves evolve to adapt to the appearance of bacterial resistance as part of a co-evolutionary arms race. Several studies have shown that phage resistant mutants do indeed appear during the course of phage therapy in farm animal and clinical trials; however, there has been success in overcoming this problem by using different phage cocktails until complete eradication of the infection (Oechslin, 2018). In addition to phage resistance development, phage-mediated horizontal gene transfer should be avoided to prevent the spread of antibiotic resistance and virulence determinants. Taking into account all these potential problems, it appears that bacteriophages, despite being a “green solution,” should be inactivated before their release outside industrial settings and farms. Of note, phages are frequently susceptible to sanitizers commonly used in the industry (Agún et al., 2018), as well as some environmental factors like pH, temperature, etc. (Jonczyk et al., 2011; Ly-Chatain, 2014), although they tend to be overall more resistant to environmental challenges than bacteria are. Nonetheless, phage inactivation can prove to be challenging following certain applications, such as crop treatment (Jones et al., 2012) or infection control in aquaculture (Richards, 2014). However, it must be noted that phage persistence in some environments is severely limited by UV-light inactivation, as is the case of the phyllosphere (Jones et al., 2012) or in aquaculture settings (Duarte et al., 2018). To overcome this limitation, some authors have recommended the application of bacteriophages at the end of the day or at night (Duarte et al., 2018). Overall, additional research is still required to examine not only the effectiveness of phages as antimicrobials, but also their persistence in the environment and the emergence of bacterial resistance in field trials.

As it can be gathered from all the above information, our knowledge about bacteriophages and their prospective use is still fairly limited. This leads funding agencies, companies and consumers to feel distrust of these new strategies, although they would be considered eco-friendly. Even for large companies, which are more suitable candidates to invest in this research, the limitations in intellectual property protection of bacteriophages (since they are isolated from nature) is a serious drawback and the implementation of the Nagoya Protocol could further complicate the matter. While a huge effort is being made from scientific institutions by requesting a change in the current path toward approval of phage products for human therapy applications (Verbeken et al., 2014; Kutter et al., 2015), there is no EU regulation allowing the use of phages as disinfectants or as prophylactic/treatment of infectious diseases in animals and plants. In the US, however, the USDA allows the application of phages to livestock animals prior to slaughtering as long as they meet the FSIS Directive 7120.1. Similarly, other countries such as Israel, Canada, Switzerland, Australia and New Zealand have adopted similar regulations.

With “a priori” fewer problems to reach the market, phage lytic proteins are taking a strong impulse in recent years. In fact, there is a significant body of work on endolysins against both Gram-positive and Gram-negative bacteria, and several proteins against *S. aureus* are now being assayed in human clinical trials (Gerstmans et al., 2017; Gutiérrez et al., 2018). These and other proteins in preclinical studies are expected to be commercialized in the next few years, and might be used in veterinary applications. In fact, the effectiveness of endolysins to treat, for instance, staphylococcal cow mastitis was revealed in a recent study carried out by Fan et al. (2016). Similarly, endolysins have been proven to be effective against the main farm animal pathogens such as *C. perfringens* (Swift et al., 2015), *Salmonella* (Rodríguez-Rubio et al., 2016) and *Streptococcus suis* (Wang et al., 2009) and even the bee pathogen *Paenibacillus larvae* (Oliveira et al., 2015). Phage lytic proteins are also promising adjuvants for classical disinfectants for surface decontamination (Gutiérrez et al., 2016). However, there are three main challenges that should be addressed before the implementation of endolysins in the veterinary sector. For instance, the immunogenicity of phage-derived proteins might result in decreased efficacy as well as in an undesirable immune response (Jun et al., 2014). Nevertheless, this issue can be solved by protein modification (Filatova et al., 2016). Another difficulty is the cost associated to endolysin production. Remarkably, new cost-effective and sustainable strategies to scale up endolysin production have been developed. For instance, recent work published by Stoffels et al. (2017) shows the suitability of a unicellular alga, *Chlamydomonas reinhardtii*, to express phage lytic proteins. Finally, the current regulatory pathways need to be adapted for these new substances. Nevertheless, approval should be more straightforward for endolysins than for phages, as multitude of therapeutic proteins are currently on the market (Dimitrov, 2012).

In summary, we currently have enough expertise and equipment to tackle the technical issues that limit the application of phage therapy. Therefore, it is only a matter of time that these drawbacks can be overcome. In turn, this information should pave the way for regulatory approval by filling the gaps regarding the efficacy and safety of these new antimicrobials. Meanwhile, we should realize that phage therapy does not necessarily have to be identical to antibiotic therapy; therefore, the peculiarities of this new bacteria control strategy may require a different approach. Given the impotence of clinicians to treat patients suffering from antibiotic-resistant infections, if the price to pay is more in-depth and thorough research, the effort will be well worth it.

## Author contributions

PG conceived and designed the work. PG, LF, DG, and AR wrote the paper.

### Conflict of interest statement

The authors declare that the research was conducted in the absence of any commercial or financial relationships that could be construed as a potential conflict of interest.
